# Cryptococcal Endocarditis in Humans—A Narrative Review

**DOI:** 10.3390/pathogens14060547

**Published:** 2025-05-31

**Authors:** Petros Ioannou, Afroditi Ziogou, Alexios Giannakodimos, Ilias Giannakodimos, Andreas G. Tsantes, George Samonis

**Affiliations:** 1 School of Medicine, University of Crete, 71003 Heraklion, Greece; 2 Department of Medical Oncology, Metaxa Cancer Hospital of Piraeus, 18537 Piraeus, Greece; aziogou@yahoo.com; 3 Department of Cardiology, Tzaneio General Hospital of Piraeus, 18537 Piraeus, Greece; 4 Department of Urology, Attikon General Hospital of Athens, 12462 Athens, Greece; iliasgiannakodimos@gmail.com; 5 Laboratory of Hematology and Blood Bank Unit, “Attikon” University Hospital, School of Medicine, National and Kapodistrian University of Athens, 12462 Athens, Greece; andreas.tsantes@yahoo.com; 6 Microbiology Department, “Saint Savvas” Oncology Hospital, 11522 Athens, Greece; 7 First Oncology Department, Metropolitan Hospital, Neon Faliron, 18547 Athens, Greece

**Keywords:** *Cryptococcus*, infective endocarditis, vegetation, fungemia

## Abstract

Background: *Cryptococcus* species constitute opportunistic fungi that seldom cause infections in individuals with competent immune systems. In the rare case of cryptococcal endocarditis, the fungus infiltrates the endocardium. This disease occurs almost exclusively in patients with active immunosuppression, implanted cardiac devices, or prosthetic valves. Objectives: This study aims to analyze all documented cases of *Cryptococcus* spp. endocarditis in humans, emphasizing the epidemiology, microbiology, clinical manifestations, therapeutic approaches, and infection outcomes. Methods: A comprehensive review was performed by searching the PubMed and Scopus databases. Results: A total of 16 studies reported data on 16 patients diagnosed with cryptococcal endocarditis. The mean patient age was 46.6 years, with males comprising 81.25% of cases. Immunosuppression was the most prevalent predisposing factor (31.25%), followed by a history of end-stage renal disease and prosthetic cardiac valves (25%). The most commonly affected intracardiac sites were the mitral (60%) and aortic valve (46.6%), while in 33.3% of cases, multiple-valve infection was observed. *Cryptococcus neoformans* was detected as the causative organism in the majority of cases (87.5%). The most frequently administered antifungal treatments included amphotericin B (87.5%) and fluconazole (43.75%), with combination therapy used in 62.5% of cases. Overall mortality was relatively high at 56.25%, with 50% of deaths directly attributed to the infection. Conclusions: Considering the ability of *Cryptococcus* spp. to induce severe systemic infections, healthcare providers should consider this pathogen in the differential diagnosis when yeast microorganisms are identified in microbiological samples. This is particularly crucial for patients with underlying comorbidities or immunodeficiency, as early recognition is crucial to ensure precise diagnosis and treatment.

## 1. Introduction

Endocarditis constitutes an infection affecting the endocardium or any implanted prosthetic material, including artificial heart valves and cardiac implantable electronic devices (CIEDs) such as pacemakers and defibrillators [1,2]. This disease is associated with high rates of complications and mortality. Traditionally, infective endocarditis (IE) is most commonly attributed to aerobic Gram-positive cocci, particularly streptococci, staphylococci, and enterococci, responsible for approximately 75% of cases [3,4]. Nevertheless, IE can also be caused by several fungal species, including *Cryptococcus* spp. Fungal endocarditis accounts for 2% of all IE cases; the most common pathogens causing fungal endocarditis are *Candida albicans* (24–46%), followed by *Aspergillus* (25%) [5,6]. Due to the rarity of infections caused by *Cryptococcus* spp., their specific clinical features remain insufficiently documented [7].

*Cryptococcus* species are encapsulated opportunistic fungal pathogens commonly found in the environment, particularly in soil, decomposing organic matter, and bird droppings, especially from pigeons [8,9]. While these fungi are the primary cause of cryptococcosis, they rarely infect individuals with a competent immune system. However, those with weakened immunity, such as patients with HIV/AIDS or malignancies, are at significantly higher risk [10]. Research indicates that cryptococcosis occurs in approximately 5–10% of immunocompromised individuals and can affect up to 30% of patients who are HIV-positive. In contrast, cases among immunocompetent individuals are extremely rare, estimated at roughly one per 100,000 people [9]. The primary route of entry for *Cryptococcus* spp. is through the respiratory system. Once inhaled, the fungus establishes itself in the lungs, potentially leading to cryptococcal pneumonia [7]. In some cases, it disseminates via the bloodstream, spreading to other organs and tissues and, rarely, resulting in cryptococcal endocarditis; this condition is rarely reported and occurs almost exclusively in patients with active immunosuppression, implanted cardiac devices, or prosthetic valves [11].

This narrative review aims to examine all documented cases of endocarditis attributed to *Cryptococcus* species, with an emphasis on its epidemiology, clinical manifestations, microbiological characteristics, therapeutic strategies, and outcomes.

## 2. Materials and Methods

### 2.1. Search Strategy and Inclusion and Exclusion Criteria

This narrative review aims to collect and showcase all available published data related to endocarditis caused by *Cryptococcus* species in humans. The primary aim was to examine the mortality rates and epidemiological patterns associated with this type of infection. Secondary objectives included detailing the exact site of infection, offering comprehensive clinical profiles of the affected patients, and summarizing microbiological findings as well as treatment regimens used for *Cryptococcus* spp. endocarditis. Two researchers (A.Z. and A.G.) independently performed a literature search in PubMed/Medline and Scopus databases for articles on *Cryptococcus* spp. endocarditis published up to 15 December 2024 using a predefined strategy. The keywords utilized for this search included “*Cryptococcus*” AND “endocarditis”. Any disagreements during the review process were resolved with input from a senior investigator (P.I.). The inclusion criteria for this review encompassed studies featuring original data, including case series, case reports, and cohort studies, that addressed the epidemiology and clinical outcomes of *Cryptococcus* spp. endocarditis in humans. Only English-language publications were considered eligible. Excluded from the review were systematic reviews, narrative reviews with aggregated data, animal studies, and articles without full-text availability or sufficient information on patients’ mortality and epidemiology. To enhance thoroughness, the references of all included studies (Table S1) were examined to identify any additional relevant publications that might have been overlooked during the initial search.

### 2.2. Data Extraction and Definitions

Data extracted from each included study comprised the publication year, article type, country of origin, patient demographics (age and gender), relevant medical history, microbiological characteristics, and details of the infection. These included the specific valve affected, diagnostic approach, all associated complications, identified pathogens, antimicrobial resistance patterns, treatment regimens, and clinical outcomes (survival or mortality). The relationship between mortality and the initial infection was recorded based on the authors’ observations in each study.

## 3. Results

### 3.1. Included Studies’ Characteristics

**Figure 1:**
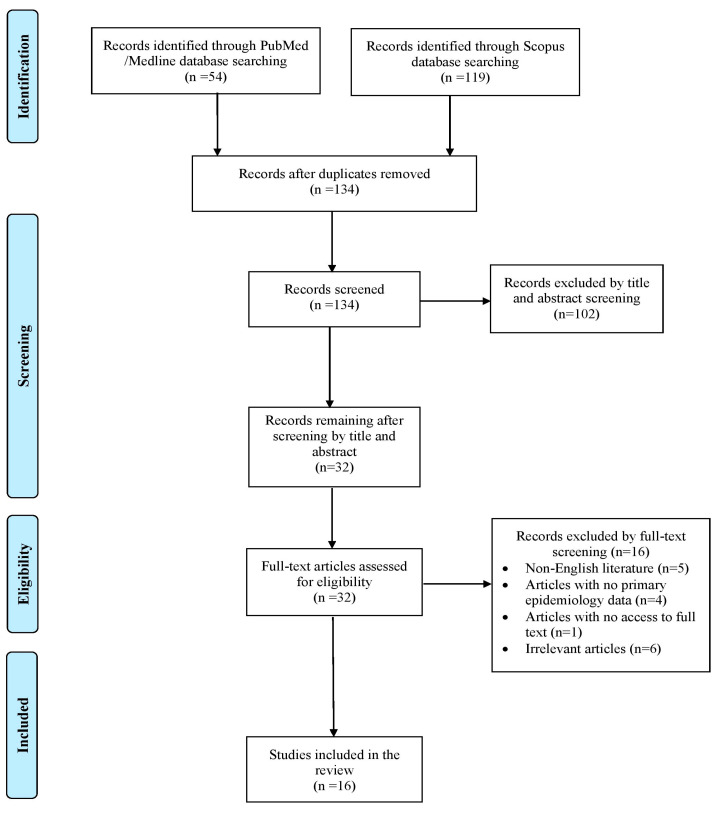
Trial flow of this narrative review.

A total of 173 articles were identified through searches in the PubMed and Scopus databases. After removing duplicates, screening records, and applying the snowballing method, only 16 articles met the inclusion criteria and were included in the final analysis [7,10,11,12,13,14,15,16,17,18,19,20,21,22,23,24]. These studies collectively reported on 16 patients. A flow diagram outlining the selection process is shown in Figure 1.

Across the included cases, 10 (62.5%) occurred in the USA, 5 (31.25%) in Asia, and only 1 (6.25%) in Europe. All selected articles were case reports.

### 3.2. Epidemiology of Cryptococcal Endocarditis

The mean age of patients diagnosed with *Cryptococcus* spp. endocarditis was 46.66 years (ranging from 4 to 74), with males comprising the great majority of the cases (81.25%; 13 patients). Regarding medical history and risk factors, five patients (31.25%) were immunosuppressed. Among these patients, three presented with hematologic malignancies, one with systemic lupus erythematosus nephritis, and one with lung transplantation due to idiopathic interstitial pneumonia. Additionally, four individuals (25%) were diagnosed with end-stage renal disease or had a prosthetic cardiac valve. In all four cases (100%) the prosthetic cardiac valve was metallic, while the time after valve replacement varied from 2 weeks to 2 years. Three cases (18.75%) had a history of malignancy or rheumatic heart disease. Of note, in all three patients (100%) the type of malignancy was hematological, while one was receiving chemotherapy (6.25%). Additionally, two patients (12.5%) underwent cardiac surgery within the past four months, were intravenous drug users (IVDUs), or had received antimicrobial agents within the last three months. Other risk factors, such as the presence of central venous catheters (CVCs) or cardiac implantable devices (CIDs), as well as previous episodes of infective endocarditis, were reported in one case (6.25%) each. Table 1 provides a detailed overview of the demographic and clinical features of all *Cryptococcus* spp. endocarditis cases.

### 3.3. Microbiology and Diagnosis of Cryptococcus spp. Endocarditis

Cryptococcal endocarditis was polymicrobial in two cases (12.5%), with blood cultures yielding *Corynebacterium striatum* in the first and *Streptococcus viridians* in the second case. Concomitant infections were observed in seven patients (43.75%) and most commonly involved the urinary tract (in four of these cases; 57.14%), followed by the respiratory system in three cases (42.85%). Of note, disseminated candidiasis and oral infection with HSV1 were noted in one case (14.28%) each. *Cryptococcus neoformans* was the isolated species in 14 of the included cases (87.5%), while in two patients (12.5%), the species responsible for the infection was not reported. None of the isolated species exhibited resistance to antifungal agents.

The diagnosis of IE was based on Duke’s criteria in 75% (12/16 cases), while transthoracic echocardiography and transesophageal echocardiography were indicative of the disease in 50% (8/16 cases) and 43.75% (7/16), respectively. Infected valve histology and culture were positive for *Cryptococcus* spp. in 31.25% (5/16 cases) and 18.75% (3/16 cases) respectively, while diagnosis was made by autopsy in 18.75% (3/16 cases). Positive blood cultures were obtained from 87.5% (14/16 cases) of cases. Notably, in one case, the pathogen was isolated from a brachial artery thrombus culture. Moreover, positive cryptococcal antigen was reported in 66.6% (8/12 patients with available data), while in three patients (18.75%), cerebrospinal fluid culture was indicative of the pathogen. Initial misdiagnosis of the disease occurred in 50% (8/16 cases) and most commonly included bacterial endocarditis (in 50%; 4/8 cases).

### 3.4. Clinical Characteristics of Cryptococcal Endocarditis

The infection most frequently involved the mitral valve in 60% (9/15 cases, with available data), followed by the aortic in 46.6% (7/15 cases) and the tricuspid in 13.3% (2/15 cases). No cases of infection of the pulmonary valve were detected. Cardiac implantable electronic device infection was noted in one case (6.25%). Multiple-valve involvement was observed in 33.3% (5/15 cases). Concomitant involvement of the CNS was detected in nine patients (56.25%). The most commonly reported clinical symptoms were fever in 78.57% (11/14 cases), organ dysfunction in 68.75% (11/16 cases), embolic phenomena in 60% (9/15 cases), heart failure in 53.84% (7/13 cases), need for intensive care unit (ICU) admission in 35.71% (5/14 cases), and shock in 6.3% (2/16). More specifically, regarding the embolic phenomena, the CNS was affected in 44.4% (4 out of 9 cases), the spleen in 33.3% (3 out of 9 cases), and the lungs in 22.2% (2 out of 9 cases). Symptoms’ duration ranged from acute onset in three patients (20%, with available data) to 4 months (in one individual) before the establishment of final diagnosis. Table 2 presents the clinical characteristics of patients with IE and the definitive treatment provided.

### 3.5. Treatment and Outcomes of Cryptococcal Endocarditis

Table 2 summarizes the definitive treatment options for all patients diagnosed with cryptococcal endocarditis. The median duration of treatment among surviving patients was 8 weeks (ranging from 2 to 36). The most frequently administered antifungal agent was amphotericin B in 14 patients (87.5%), followed by fluconazole in 7 (43.75%), and flucytosine and micafungin, administered in 4 patients (25%) each. Two patients (12.5%) received voriconazole, while only one (6.25%) received caspofungin. Notably, 10 individuals (62.5%) received antifungal combination treatment. Only one patient did not receive treatment with antifungals; of note, the clinical outcome for this particular patient was death due to the infection. A combination of surgical intervention and antifungal therapy was utilized in 25% (four cases) and mainly consisted of infected valve removal and replacement. Interestingly, 11 patients (68.75%) received antimicrobial agents; in two individuals (12.5%) these agents were administered as treatment of concomitant infections, while in the remaining nine patients (56.25%), they were administered due to misdiagnosis of the disease, mainly for bacterial endocarditis.

The overall mortality rate was 56.25% (9 out of 16), with 50% of deaths (eight patients) directly linked to the IE episode. In one patient, death was attributed to complications arising from non-adherence to prescribed hemodialysis sessions. Amongst the 12 patients who were not subjected to surgical approaches, only 4 survived (33.33%).

## 4. Discussion

This review examined the clinical profiles of patients diagnosed with IE caused by *Cryptococcus* species, drawing data from multiple studies to provide a comprehensive overview of their epidemiology, microbiology, clinical presentation, treatment approaches, and outcomes. The mitral valve was the most frequently affected intracardiac site, followed by the aortic valve. Fever, organ dysfunction, embolic events, and heart failure were the predominant clinical manifestations. Regarding definitive antifungal therapy, amphotericin B and fluconazole were the most frequently administered treatment options. Additionally, the review highlights the high overall mortality rate linked to *cryptococcal* IE.

The limited number of documented cases of *Cryptococcus* spp. IE in the available literature makes it challenging to establish precise epidemiological data. In this review, most reported cases involved male patients, with a mean age of 46.6 years. Intriguingly, the majority of cases were reported in the USA; this greater prevalence observed in the USA may be attributed to more advanced clinical surveillance systems in these regions. The complete lack of reported cases in Africa may indicate that these infections may not have a strong correlation with suboptimal living conditions. Moreover, it may relate to the health care system, infrequent valve replacement surgeries as well as diagnostic limitations. Nevertheless, the limited number of studies conducted globally hinders the ability to establish definitive epidemiological patterns for *cryptococcal* IE.

Previously known as European blastomycosis, torulosis, or Busse–Buschke disease, cryptococcosis is an opportunistic infection caused by species of *Cryptococcus* [25]. Described for the first time in 1894, *Cryptococcus* spp. constitute a dimorphic, heterobasidiomycetous, encapsulated fungus that exists as both an environmental organism and an opportunistic pathogen. It is commonly found in avian droppings, soil, and trees [26]. Various environmental sources contribute to community exposure to *Cryptococcus neoformans*; research has proven that the fungus has been detected in 11.5% of bird droppings, 8.5% of tree surface samples, 6.7% of soil specimens, and 10% of waste disposal areas in Kenya [27]. Over 95% of cryptococcal infections are caused by *Cryptococcus neoformans*, which predominantly leads to systemic cryptococcosis in immunocompromised individuals. The remaining cases are attributed to *Cryptococcus gattii* [28,29]. Though once thought to be limited to tropical and subtropical regions, *C. gattii* has now been reported worldwide, thriving in trees and tree hollows [30]. While the inhalation of cryptococcal spores or desiccated yeast cells is typically asymptomatic in the general population, it can result in pulmonary or systemic cryptococcosis in those with weakened immune systems. The infection usually enters the body via the lungs, often leading to pneumonia and meningitis [7]. The fungus may remain dormant depending on the host’s immune status, and infection may later develop through reactivation of a latent pulmonary infection, similar to tuberculosis [31]. In rare cases, *Cryptococcus* may affect the heart, involving the pericardium, myocardium, and endocardium; cryptococcal endocarditis constitutes an uncommon condition that typically originates from a primary lung infection, spreading through the bloodstream [10,15].

The virulence of *Cryptococcus* is primarily determined by three fundamental processes: its ability to adapt to the host environment, evade immune responses, and generate various virulence factors. Although similar mechanisms exist in other fungal pathogens, *Cryptococcus* species are particularly notable for their diverse strategies in host adaptation, immune evasion, and virulence factor production [32]. In order to survive within the host, *Cryptococcus* adjusts to environmental pressures such as temperature fluctuations, nutrient availability, pH variations, and oxidative stress. This adaptation relies on metabolic shifts and the activation of signaling pathways, including mitogen-activated protein kinases (MAPKs) [33]. A crucial aspect of its pathogenicity is its capability to grow at 37 °C, a temperature that most environmental fungi do not tolerate. This thermal adaptability is believed to play a significant role in its ability to infect immunocompromised individuals [34]. In addition to its thermal resilience, *Cryptococcus* produces various enzymes that enhance its virulence, including proteases, lipases, and urease. Urease, in particular, facilitates nitrogen metabolism by hydrolyzing urea into ammonia and carbon dioxide. The high production of this enzyme by *Cryptococcus* is not only vital for its survival but also serves as a diagnostic marker for cryptococcal infections [35]. Moreover, two critical virulence factors of *Cryptococcus* include the polysaccharide capsule and melanin production. The capsule shields the pathogen from phagocytosis and environmental stressors like dehydration and oxidative damage and also modulates the host’s immune response [36,37]. Melanin contributes to its pathogenicity by protecting the fungus from oxidative stress, radiation, and high temperatures. Mutants lacking melanin exhibit diminished virulence, highlighting its importance. Finally, melanin can reduce the effectiveness of antifungal treatments, further enhancing the pathogen’s ability to persist within the host [38,39].

In the present review, immunosuppression was identified as the most prevalent predisposing risk factor. The immune system effectively clears most cases of *Cryptococcus neoformans* in healthy individuals. Consequently, the infection predominantly occurs in immunocompromised individuals and is a significant opportunistic infection associated with HIV/AIDS, particularly in developing countries [40]. Among the five immunosuppressed patients recorded in the present review, three were diagnosed with malignancies, one underwent lung transplantation, and another was diagnosed with systemic lupus erythematosus (SLE). In organ transplant patients, immunosuppression and infection with *Cryptococcus* spp. may result from immunosuppressive drug treatment such as steroid therapy [10]. A case report by Yavari et al. presented a patient with SLE and end-stage renal disease requiring a permanent hemodialysis catheter. The presence of this catheter, combined with prolonged immunosuppression, may have contributed to the acquisition of *Cryptococcus* [16]. This was the only patient on hemodialysis among the five reported patients with ESRD. In accordance with previous studies reporting cryptococcal cardiac involvement in patients with prosthetic valves, the findings of this review suggest that 25% of cases had prosthetic valves, particularly metallic ones [41]. The incidence of cryptococcal IE has risen over the past decades, mainly due to advancements in medical and surgical treatments, especially prosthetic valve surgery. The risk of infection is highest within the first year after surgery, affecting between 1% and 3% of patients [42,43]. Regarding the four cases of cryptococcal IE described in the present review, infection developed 2 weeks after valve replacement in one patient, while in two other patients, infection occurred 1 and 3 years after replacement, respectively. No data are available concerning the time between valve replacement and cryptococcal IE regarding the fourth case. In immunosuppressed individuals with prosthetic cardiac valves, the risk of cryptococcal IE is significantly elevated [22].

History of malignancy as well as rheumatic fever constitute additional important predisposing factors for cryptococcal IE, noted in 18.75% of the included patients of this review. Interestingly, all patients with cancer diagnosed with cryptococcal IE had hematological malignancies, such as leukemia, either acute or chronic. One pediatric patient, diagnosed with acute leukemia, received chemotherapy [17]. In regards to rheumatic fever, it is an inflammatory disease that can develop as a complication of untreated or inadequately treated streptococcal throat infection caused by Group A *Streptococcus*. This condition may facilitate cryptococcal growth by causing chronic valvular damage and the requirement for valve replacement surgery using prosthetic valves; prosthetic valves exhibit a higher risk of fungal infections. Moreover, long-term antibiotic prophylaxis and steroids or immunosuppressants administered to these patients enable fungal growth [44]. Recent cardiac surgery is another risk factor for the development of cryptococcal IE, as the pathogen has an affinity for prosthetic materials and can form biofilms through adherence to artificial surfaces. Surgical trauma to the endocardium and valves creates a surface where *Cryptococcus* can adhere and proliferate [12]. Surgical procedures may introduce fungal spores into the bloodstream, leading to cryptococcemia and heart valve infections. Another pediatric patient had a history of recent cardiac surgery as well as rheumatic heart disease [13]. Prolonged hospitalization, use of central venous catheters, and broad-spectrum antibiotics associated with cardiac surgeries can disrupt normal immune defenses, increasing the risk of opportunistic fungal infections [11,45]. Finally, patients with IVDU or CIDs are predisposed to cryptococcal IE. In IVDU, *Cryptococcus neoformans* can enter the bloodstream through contaminated injections, leading to fungemia and subsequent endocarditis. Of note, frequent injections may provoke recurrent bacteremia, leading to chronic damage to the heart valves [11,15].

Cryptococcal species have the potential to affect various organs, with the central nervous system being the most frequently involved, causing meningitis in nearly 75% of cases. Pulmonary and skin infections are also common and can advance to systemic disease. However, IE due to this fungus is exceptionally rare and has mainly been reported in only a few case studies [18]. Cryptococcal endocarditis does not exhibit distinct clinical features that distinguish it from other causes of endocarditis. Typical presenting symptoms include fever, respiratory distress, and even delirium [11]. This is in accordance with the findings of the present review, in which fever constituted the most frequent clinical presentation, observed in 78.57% of the included patients. More complicated clinical manifestations include embolic phenomena, which were the most frequently observed complications in this review, along with heart failure and the need for ICU admission. Notably, the majority of the embolic phenomena involved primarily the CNS and, secondarily, the spleen. Symptoms may persist for up to four months before the final diagnosis is confirmed [12]. The most frequently infected intracardiac sites were the mitral valve in 60% followed by the aortic and the tricuspid. Interestingly, both cases of tricuspid valve IE had injected drugs intravenously and also had cryptococcal pneumonia. Although most cases of cryptococcal endocarditis are limited to a single intracardiac site, infection of multiple valves simultaneously has been reported; in this review, 33.3% of patients exhibited multiple-valve infection. Of note, both patients with right-sided endocarditis also had left-sided valve involvement. This dual involvement is uncommon but not unprecedented, particularly in patients with a history of intravenous drug use, which can lead to hematogenous seeding of both right and left heart valves. A possible hypothesis for this is that concurrent left-sided valve infection could be due to a combination of factors, including prolonged bacteremia, impaired host immunity, and direct extension of the infection from the right to the left side due to cardiac shunting or valvular damage. In all cases of cryptococcal endocarditis, *Cryptococcus neoformans* was identified as the pathogen responsible. The misdiagnosis rate for cryptococcal endocarditis is relatively high due to its non-specific clinical features; it is commonly mistaken for bacterial endocarditis, respiratory infection, and, in one case, even for atrial myxoma [21,23]. The present review also highlights high misdiagnosis rates, with 50% of patients initially being initially misdiagnosed. Among them, 50% (four cases) were mistakenly diagnosed with bacterial endocarditis.

Detecting *Cryptococcus* species using conventional diagnostic methods remains demanding; early clinical suspicion remains essential for an effective diagnostic approach [14]. Historically, the diagnosis of fungal endocarditis has relied on the Duke Criteria, which may overlook infections caused by atypical microorganisms. Therefore, it is essential for clinicians to maintain a high level of suspicion in patients with relevant risk factors [46]. Blood culture constitutes a crucial diagnostic test for IE and, although negative blood cultures present a diagnostic challenge, their sensitivity for cryptococcal endocarditis is still relatively high. In cases of cryptococcal endocarditis, most blood cultures yield positive results [11]. However, fungal culture is often slow and may lack sensitivity. Another traditional method aiding the diagnosis is the cryptococcal capsular polysaccharide antigen (CrAg) test. The CrAg test is the most sensitive and specific diagnostic tool currently available, with sensitivity and specificity both exceeding 96%. Despite its advantages, it has some drawbacks, such as an inability to confirm active infection, detect antigen-deficient strains, or distinguish between different *Cryptococcus* species [47]. Serum cryptococcal antigen (SCA) has demonstrated high sensitivity and specificity in diagnosing cryptococcemia and meningitis, and therefore, it should also have a high sensitivity for cryptococcal endocarditis [48]. In the present review, 66.6% of the included cases of cryptococcal endocarditis tested positive for SCA. The literature suggests that lumbar puncture should be conducted in order to rule out CNS involvement, as cryptococcemia is strongly associated with meningitis development [11,49]. Echocardiography was widely applied in the majority of patients in the present review. Of note, transthoracic echocardiogram (TTE) detected vegetations in 50%, while transesophageal echocardiogram (TEE) detected them in 43.75% of patients. The sensitivity of TTE and TEE for cryptococcal endocarditis may actually be high. Nonetheless, negative echocardiography results for IE can occur in about 15% of cases. Moreover, guidelines recommend repeating TEE if IE is strongly suspected [50]. Valve histology of the removed valves was successfully conducted in 31.25% of the included patients and disclosed encapsulated single budding yeasts identified as *Cryptococcus* spp [13].

The antifungal treatment for cryptococcal IE is generally based on approaches used for other forms of disseminated cryptococcosis and includes a combination of antifungal treatment and surgical intervention [10]. According to the guidelines for the management of cryptococcosis, first-line treatment for disseminated cryptococcosis includes liposomal amphotericin B in combination with flucytosine as induction therapy, particularly for patients who are not infected with HIV [51,52]. The recommended duration of induction treatment is a minimum of 2 weeks, followed by a consolidation phase comprising fluconazole for 8 weeks [52]. Furthermore, in patients with a persistent immunocompromised state, maintenance treatment with lower doses of fluconazole is recommended. If the patient presents with a severe pulmonary co-infection or CNS infection, the induction treatment with liposomal amphotericin B in combination with flucytosine may be extended in duration [10,52]. It is also worth mentioning that a few of the reported cases received an echinocandin; these agents have negligible activity against cryptococci and are mainly recommended for the treatment of candida subspecies. Information regarding surgical intervention is largely drawn from other types of fungal endocarditis. Typically, surgery is considered due to the high mortality rate associated with this infection, with a low threshold for surgery [52,53]. As a result, cryptococcal endocarditis should be regarded as an indication for a combined treatment approach, involving both antifungal therapy and surgical debridement of the infected valves [54]. However, comorbidities often preclude surgery. For this reason, only 25% of the included patients in this review were subjected to surgery; in three patients, the surgical management consisted of removal and replacement of the infected valve, while one patient underwent intraarterial vegetation removal [17]. Moreover, in the management of cryptococcal infections, it is generally recommended to reduce immunosuppression. This must be performed carefully, as alongside *Cryptococcus* treatment, this can potentially lead to immune reconstitution inflammatory syndrome (IRIS). This may be attributed to a shift occurring from a dominant T-helper 2 (Th2) response, fostered by immunosuppression and the polysaccharide of *C. neoformans*, to a T-helper 1 (Th1) response, and it may be triggered by both the reduction in immunosuppression and the initiation of treatment for *Cryptococcus* [55].

The mortality rate of cryptococcal endocarditis remains high, potentially due to factors such as underlying conditions, delayed diagnosis, and decisions regarding the need for surgery [7]. An overall mortality rate of 56.25% was observed among the patients in this review. However, many of the available reports are outdated, and it is possible that the mortality rate for cryptococcal endocarditis has improved with advancements in diagnosis and treatment in recent times.

This review is subject to a number of limitations. The literature search may not have retrieved all pertinent research on epidemiology and mortality, as some studies may have been overlooked due to the search strategy. The analysis was restricted to case reports and case series, which rely on accurate reporting to ensure validity. Additionally, incomplete data from several studies limited the analysis, restricting it to the available information. Consequently, findings were only derived from studies with comprehensive data. Furthermore, in cases reporting concurrent bacterial and cryptococcal endocarditis, although cryptococcosis most likely involved the heart, the potential contribution of bacterial endocarditis cannot be fully excluded and remains a limitation. Finally, studies published in languages other than English were not included.

## 5. Conclusions

This narrative review provides valuable perspectives into the epidemiology, clinical features, microbiology, treatment, and clinical outcomes of cryptococcal endocarditis, emphasizing its pathogenic potential. *C. neoformans* emerged as the most commonly identified species, with the mitral valve being the most frequently affected. While no formal treatment guidelines have been established, a combination of amphotericin B, flucytosine, and, in some cases, fluconazole was the most commonly used antifungal regimen. Timely initiation of antifungal therapy is critical for effective management. The immune status of patients plays a crucial role in determining clinical outcomes. This review of rare cases highlights the challenges in diagnosing fungal cardiac infections and underscores the importance of prompt medical and surgical treatment to manage patients with cryptococcal endocarditis. Although this review is subject to certain limitations, it highlights the importance of conducting further longitudinal studies and controlled research to deepen the understanding of *Cryptococcus* spp. endocarditis and create future treatment strategies.

## Figures and Tables

**Table 1:** Characteristics of patients with cryptococcal endocarditis.

Characteristic	All Patients (*n* = 16) *	Survived (*n* = 7) *	Died (*n* = 9) *
Age, years, median (IQR)	50	26	53.5
Male gender, *n* (%)	13 (81.25)	7 (100)	6 (66.67)
Predisposing factors			
Immunosuppression, *n* (%)	5 (31.25)	2 (28.57)	3 (33.3)
End-stage renal disease, *n* (%)	4 (25)	1 (14.28)	3 (33.3)
Prosthetic cardiac valve, *n* (%)	4 (25)	1 (14.28)	3 (33.3)
Malignancy, *n* (%)	3 (18.75)	1 (14.28)	2 (22.2)
Rheumatic heart disease, *n* (%)	3 (18.75)	1 (14.28)	2 (22.2)
Recent cardiac surgery, *n* (%)	2 (12.5)	1 (14.28)	1 (11.1)
IVDU, *n* (%)	2 (12.5)	1 (14.28)	1 (11.1)
Recent antibiotic administration, *n*(%)	2 (12.5)	2 (28.57)	0
Concomitant infection, *n* (%)	7 (43.75)	2 (28.57)	5 (55.56)
Clinical characteristics			
Fever, *n* (%)	11/14 (78.57)	4/5 (80)	7 (77.78)
Sepsis, *n* (%)	4/14 (28.57)	0	4 (44.44)
Treatment			
Amphotericin, *n* (%)	14/16 (87.5)	6 (85.71)	8 (88.88)
Fluconazole, *n* (%)	7/16 (43.75)	3 (42.85)	4 (44.44)
Micafungin, *n* (%)	4/16 (25)	2 (28.57)	2 (22.2)
Flucytosine, *n* (%)	4/16 (25)	2 (28.57)	2 (22.2)
Caspofungin, *n* (%)	1/16 (6.25)	1 (14.28)	0
Antifungal Combination	10/16 (62.5)	5 (71.42)	5 (55.5)
Outcomes			
Deaths due to infection, n (%)	8/16 (50)	NA	NA
Deaths overall, n (%)	9/16 (56.25)	NA	NA

NA: Not applicable, IVDU: intravenous drug user, IQR: interquartile range; *: data are among the number of patients mentioned on top unless otherwise described.

**Table 2:** Clinical manifestations and treatment of patients with cryptococcal endocarditis in all patients and according to patient outcomes.

Characteristic	All Patients (*n* = 16) *	Survived (*n* = 7) *	Died (*n* = 9) *
Fever, *n* (%)	5/15 (33.3)	4/5 (80)	7 (77.77)
Embolic phenomena, *n* (%)	9/15 (60)	4 (57.14)	5/8 (62.5)
Heart failure, *n* (%)	7/13 (53.84)	2 (28.57)	5/6 (83.3)
Need for ICU, *n* (%)	5/14 (35.71)	1 (14.28)	4/7 (57.14)
Sepsis, *n* (%)	4/14 (28.57)	0	4/7 (57.14)
Shock, *n* (%)	2 (12.5)	0	2 (22.2)
Paravalvular abscess, *n* (%)	1 (6.25)	0	1 (11.1)
Treatment			
Amphotericin, *n* (%)	14 (87.5)	6 (85.71)	8 (88.88)
Fluconazole, *n* (%)	7 (43.75)	3 (42.85)	4 (44.4)
Flucytosine, *n* (%)	4 (25)	2 (28.57)	2 (22.2)
Micafungin, *n* (%)	4 (25)	2 (28.57)	2 (22.2)
Voriconazole, *n* (%)	2 (12.5)	2 (28.57)	0
Caspofungin, *n* (%)	1 (6.25)	1 (14.28)	0
Surgical Management, *n* (%)	4 (25)	3 (42.85)	1 (11.1)

*: Data are among the number of patients mentioned at the top unless otherwise described.

## Data Availability

Not applicable.

## Supplementary Materials

The following supporting information can be downloaded at: https://www.mdpi.com/article/10.3390/pathogens14060547/s1, Table S1: Characteristics of all included studies.
