# Supplementary material for: Cryptococcal Endocarditis in Humans—A Narrative Review
Source: Pathogens. 2025 May 31;14(6):547. doi: 10.3390/pathogens14060547 (PMC12195974; doi:10.3390/pathogens14060547)
Supplement: Supplementary file 1 [file pathogens-14-00547-s001.zip › pathogens-3650860-supplementary.pdf]

Author, Year	Number of Patients	Gender	Age (Years)	Comorbidities	Isx	Imaging method	Site of Infection	Valve type	Treatment	Surgery	Fatality
Nakajima et al., 2019	1	Male	72	ICD, HTN, T2DM, CIED, Hepatitis B	-	TEE	CIED	Native	Amphotericin, Fluconazole, Micafungin	-	Yes
Fountain et al., 2021	1	Male	65	Lung transplantation	+	TEE	Aortic valve	Native	Amphotericin, Flucytosine, Voriconazole, Micafungin	-	No
McGuire et al., 2022	1	Female	65	IVDU, ESRD, COPD, heart failure	-	TTE	Aortic/ Tricuspid valve	Native	Amphotericin, Flucytosine, Micafungin	-	Yes
Alhaji et al., 2011	1	Male	41	ESRD, HTN,	-	TTE, TEE	Aortic valve	Bioprosthetic	Amphotericin	-	No
Blanc et al., 1996	1	Male	12	RHD history, recent cardiac surgery*	-	TTE	Mitral valve	Native	Amphotericin, Fluconazole	+	No
Elkhatib et al., 2022	1	Male	NR	CLL, HTN, T2DM, CVA	+	TTE, TEE	Aortic valve	Native	Amphotericin, Fluconazole	-	Yes
Roy et al., 2018	1	Male	26	IVDU	-	TTE, TEE	Mitral/ Tricuspid valve	Native	Amphotericin, Fluconazole	-	No
Yavari et al., 2023	1	Female	37	CVC, ESRD, SLE nephritis	+	TTE, TEE	Mitral valve	Native	Amphotericin, Fluconazole, Flucytosine	+	Yes
Kowatari et al., 2019	1	Male	4	ALL	+	TTE	Mitral valve	Native	Voriconazole, Micafungin, Caspofungin	+	No
Li et al., 2020	1	Male	50	CVA, Recent Surgery, Hepatitis B	-	TTE	Aortic valve	Native	Amphotericin, Fluconazole, Flucytosine	+	No

Colmers et al., 1967	1	Male	55	Bright's disease	-	NR	NR	Native	Amphotericin	-	No
Benerjee et al., 1997	1	Male	48	recent cardiac surgery*, RHD history	-	NR	Mitral valve	Metallic	Amphotericin	-	Yes
Lombardo et al., 1957	1	Male	44	RHD history, recent bone injuries	-	Autopsy	Aortic/ Mitral valve	Native	None	-	Yes
Channell et al., 1996	1	Female	74	ESRD, HTN, T2DM	-	TEE	Mitral valve	Metallic	Amphotericin, Fluconazole	-	Yes
Child et al., 1979	1	Male	56	Hematologic malignany	+	Autopsy	Mitral valve	Native	Amphotericin	-	Yes
Harford et al., 1974	1	Male	51	Previous IE	-	Autopsy	Aortic/ Mitral valve	Metallic	Amphotericin	-	Yes

Supplementary Table S1. Characteristics of all included studies

ISx: Immunosuppression, ICD: Ischemic Heart Disease, HTN: Hypertension, T2DM: Type 2 Diabetes Mellitus, CIED: cardiac implantable electronic device, IVDU: intravenous drug user, ESRD: End Stage Renal Disease, COPD: Chronic Obstructive Pulmonary Disease, RHD: Rheumatic Heart Disease, CLL: Chronic Lymphocytic Leukaemia, CVA: cerebral vascular accident,CVC: Central Venous Catheter, SLE: Systemic Lupus Erythematosus, ALL: Acute Lymphocytic Leukaemia, IE: Infective Endocarditis, TEE: Transesophageal Echocardiogram, TTE: Transthoracic Echocardiogram, NR: not reported

**within the previous 4 months*
